# Evaluation and comparison of pharmacokinetic profiles and safety of two extended-release buprenorphine formulations in common marmosets (*Callithrix jacchus*)

**DOI:** 10.1038/s41598-023-38973-2

**Published:** 2023-07-22

**Authors:** Niora J. Fabian, Anthony J. Mannion, Morgan Jamiel, David. J. Anderson, Joseph E. Rower, Christopher A. Reilly, William Menegas, Sureshkumar Muthupalani, Christina Ta, James G. Fox, Robin Kramer, Jennifer L. Haupt

**Affiliations:** 1 Division of Comparative Medicine, Massachusetts Institute of Technology, Cambridge, MA USA; 2 Department of Pharmacology and Toxicology, Center for Human Toxicology, University of Utah, Salt Lake City, UT USA; 3 Simons Center for the Social Brain, Massachusetts Institute of Technology, Cambridge, MA USA; 4 StageBio, Mount Jackson, VA USA

**Keywords:** Therapeutics, Clinical pharmacology, Pharmacokinetics, Pain, Machine learning, Drug safety

## Abstract

While sustained-release buprenorphine (BSR) is used as a long-lasting opioid analgesic in common marmosets (*Callithrix jacchus*), there are no published studies on pharmaceutical-grade extended-release buprenorphine options such as Ethiqa XR (EXR) for this species. However, BSR is a compounded product and has been reported to cause injection site reactions in multiple species, including marmosets. Additionally, now with the availability of EXR, a pharmaceutical-grade veterinary product, the use of BSR in laboratory animals is not compliant with the *Guide for the Care and Use of Laboratory Animals* (*Guide*) unless scientifically justified and approved by the IACUC. We compared pharmacokinetic and safety profiles of BSR (0.15 mg/kg) and EXR (0.1–0.2 mg/kg) administered subcutaneously to adult marmosets. Blood was collected by venipuncture of the saphenous vein at multiple time points (0.25–72 h) and analyzed by liquid chromatography-tandem mass spectrometry (LC–MS/MS). EXR between 0.1 and 0.2 mg/kg resulted in a dose-dependent increase in C_max_ (1.43–2.51 ng/mL) and were not statistically different from BSR (1.82 ng/mL). T_max_, lambda_z_, and t_1/2_ were not statistically different between formulations. Mean plasma buprenorphine concentrations for BSR and EXR exceeded the therapeutic threshold (0.1 ng/mL) within 0.25 h and lasted for > 72 h. Mild sedation, but neither respiratory depression nor ataxia, was observed for both formulations. BSR injection sites had significantly higher histopathological scores compared to EXR. Video recordings for monitoring drug-induced behavioral changes showed increased animal activity levels after BSR and EXR versus saline controls. Norbuprenorphine, a buprenorphine metabolite associated with respiratory depression, was detected in the plasma after BSR and EXR administration as well as by in vitro liver microsome assays. In conclusion, we recommend using EXR over BSR as a long-lasting buprenorphine analgesic in marmosets because EXR is a pharmaceutical-grade formulation that is compliant with FDA guidelines and the *Guide* as well as exhibits comparable PK and safety profiles as BSR.

## Introduction

The common marmoset (*Callithrix jacchus*) is a New World nonhuman primate (NHP) used in neuroscience, transgenics, infectious disease, and other diverse fields of biomedical research^1–4^. Procedures outlined in IACUC approved protocols, routine veterinary care, and other clinical interventions in laboratory marmosets may require analgesia^5^. The provision of adequate analgesia is a humane necessity and is fundamental to animal research regulatory policies^6–8^.

Buprenorphine, a DEA Schedule III narcotic and partial μ-agonist, δ- and κ-antagonist, and nociceptin receptor agonist^9–11^, is the most commonly used opioid analgesic in NHPs, including common marmosets^4,12–16^. Immediate-release buprenorphine (Bup HCl) dose recommendations for marmosets range from 0.005 to 0.02 mg/kg, either alone for mild to moderate pain, or in multimodal analgesic plans for moderate-to-severe pain^4,12,14,17,18^. Two recent independent studies showed that intramuscular (IM) or subcutaneous (SQ) Bup HCl at 0.01 or 0.02 mg/kg in marmosets remained above a therapeutic plasma concentration threshold of 0.1 ng/mL for approximately 4–8 h^12,14^. This therapeutic threshold originates from buprenorphine efficacy studies in dogs^19^ and humans^10^ and has been used as a target in macaque and marmoset buprenorphine pharmacokinetic (PK) studies^15,16,20^. Based on these studies, Bup HCl may need to be administered every 4–8 h in marmosets to remain above the therapeutic threshold. Such frequent redosing may have negative effects, such as increased workload burden on veterinary staff, increased handling and stress of animals, and increased opportunities for occupational health hazards. Thus, extended-release buprenorphine formulations may be more suitable options for pain management in marmosets.

Sustained-release buprenorphine (BSR) is often used in marmosets and other NHPs as an alternative to Bup HCl for extended pain management. PK studies of BSR in macaques^16^ and rats^21^, as well as the recent study in marmosets^14^, have shown that plasma drug levels are maintained above therapeutic thresholds ≥ 3 days longer than Bup HCl. Despite these advantages, BSR is a non-pharmaceutical grade, compounded formulation that is not approved or indexed by the FDA for any species. The use of BSR may also be restricted due to institutional regulations on compounded drugs. Furthermore, BSR has also been reported to cause injection-site reactions in many species, including marmosets, macaques, mice, and rats^16,21–29^. The mechanisms of this adverse reaction is not fully understood but may be due to the polymer-encapsulated vehicle^22,24^.

Ethiqa XR (EXR) is another extended-release buprenorphine option that has been recently introduced to laboratory animal medicine. EXR is the only pharmaceutical grade, compliant with current Good Manufacturing Practices (cGMP), FDA-indexed extended-release buprenorphine analgesic indicated for the control of post-procedural pain in mice and rats for up 72 h after a single injection^30^. Due to its lipid-encapsulated (primarily cholesterol), low viscosity suspension, EXR allows gradual in situ diffusion of relatively large doses of buprenorphine to be safely administered and provides a prolonged therapeutic effect^30^. These advantages may improve buprenorphine administration in marmosets and may also mitigate the risk for injection-site reactions. Currently, there are no published studies on the PK, safety, or use of EXR in marmosets.

Marmosets are highly susceptible to significant adverse effects of buprenorphine compared to Old World NHPs such as macaques^16,17,20,31^. Respiratory depression, apnea, and death have occurred in marmosets after receiving buprenorphine at doses that are considered safe for other species, particularly when combined with alfaxalone or isoflurane^17,31^. In humans, the hepatic metabolism of buprenorphine into norbuprenorphine and subsequent glucuronidation causes respiratory depression^10^. The metabolism of buprenorphine in marmosets is unknown, but rapid production and accumulation of norbuprenorphine may account for the high risk of respiratory depression in this species.

In this study, we compared the PK profiles of single-dose BSR at 0.15 mg/kg and EXR at 0.1 to 0.2 mg/kg administered subcutaneously to adult marmosets. Due to the potential for adverse effects such as respiratory depression related to drug vehicle and buprenorphine metabolites, the safety profiles were also evaluated by direct clinical observations and machine-learning analysis of video-recorded animal activity. Additionally, BSR and EXR drug injection sites were clinically evaluated, and drug injection-site histopathology was performed in another cohort of animals. Metabolism of buprenorphine into norbuprenorphine and its glucuronide conjugates were also evaluated in plasma as well as in vitro using liver microsomes. We hypothesized that EXR would yield a similar PK profile as BSR with fewer incidences of adverse effects.

## Results

### Pharmacokinetics

**Figure 1:**
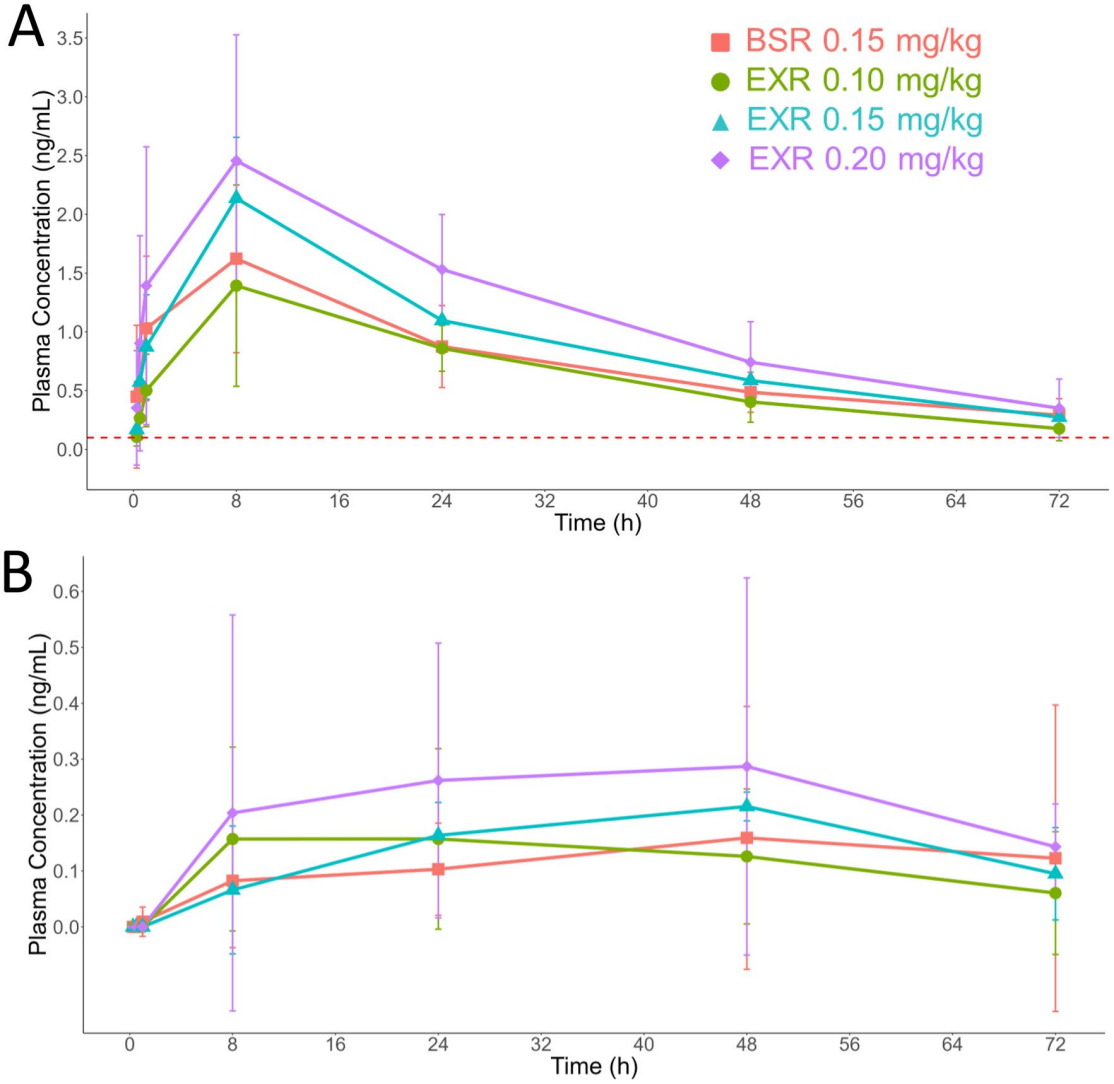
Plasma concentration–time curves of buprenorphine (**A**) and norbuprenorphine (**B**) after a single injection of BSR 0.15 mg/kg (n = 8), EXR 0.10 mg/kg (n = 6), EXR 0.15 mg/kg (n = 3), or EXR 0.20 mg/kg (n = 8). Data reported as mean ± standard deviation.

Buprenorphine plasma levels after BSR and all three doses of EXR reached the hypothesized therapeutic threshold of 0.1 ng/mL within 15 min and remained above this concentration for 72 h post-administration (Fig. 1A). Mean plasma concentrations of buprenorphine did not reach the lower limit of quantitation during the study. Thus, the time of last measurable concentration (T_last_) values were the same for all treatment groups (Table 1) due to the study design (i.e., the last study time point for all animals was 72 h). Extrapolations of the mean concentration curves remained above the 0.1 ng/mL therapeutic threshold for 129.5 h for the BSR group, 64.2 h for the EXR 0.1 mg/kg group, 104.3 h for the EXR 0.15 mg/kg group, and 87.7 h for the EXR 0.2 mg/kg group. (Supplementary Table 5) The maximum concentrations of buprenorphine detected in the plasma (C_max_) ranged from 1.4 to 2.5 ng/mL and increased in a dose-dependent manner for EXR. The time to maximum levels of buprenorphine detected in the plasma (T_max_) was reached between approximately 6–13 h and did not appear to associate with BSR or any specific EXR dose. EXR doses exhibited shorter t_1/2_ of ~ 21–24 h versus ~ 36 h for BSR. Clearance for BSR and EXR ranged from 1.7 to 2.2 L/h/kg. Despite differences in PK parameters values, none were found to be statistically significant between BSR and EXR or between EXR doses (Supplementary Table 1).

**Table 1:** PK parameters for BSR and EXR doses.

Parameter^a^	BSR 0.15 mg/kg	EXR 0.1 mg/kg	EXR 0.15 mg/kg	EXR 0.2 mg/kg
C_max_ (ng/mL)	1.82 ± 0.74	1.43 ± 0.82	2.14 ± 0.52	2.51 ± 0.97
T_max_ (h)	6.25 ± 3.24	13.33 ± 8.26	8 ± 0	10 ± 5.66
T_last_ (h)	72 ± 0	72 ± 0	72 ± 0	72 ± 0
t_1/2_ (h)	36.71 ± 17.88	21.57 ± 14.86	24.24 ± 4.74	22.71 ± 13.48
AUC_0–Tlast_ (ng × h/mL)	55.48 ± 18.52	47.02 ± 13.02	67.37 ± 2.45	86.52 ± 27.51
AUC_0–∞_ (ng × h/mL)	73.73 ± 28.85	59.28 ± 10.04	77.28 ± 1.91	105.94 ± 39.97
Clearance (L/h/kg)	2.26 ± 0.68	1.73 ± 0.34	1.94 ± 0.05	2.14 ± 0.83
k_e_ (L/h)	0.023 ± 0.011	0.041 ± 0.019	0.029 ± 0.006	0.037 ± 0.013
MRT (h)	25.2 ± 2.89	25.66 ± 6.32	24.35 ± 2.94	25.13 ± 6.05
MRT_0–∞_ (h)	50.9 ± 24.19	33.6 ± 21.66	35.03 ± 7.35	34.57 ± 18.72

^a^P-values > 0.05 for all parameter between treatment groups.

Norbuprenorphine was quantifiable in the plasma after BSR and EXR on average as early as 8 h after drug-administration and could still be quantified up to 72 h (Fig. 1B). Buprenorphine and norbuprenorphine plasma concentrations did not correlate with each other. For example, animals exhibiting similar PK profiles for buprenorphine had high variation in the levels and duration of norbuprenorphine detected (data not shown).

### Animal health and adverse effects

**Figure 2:**
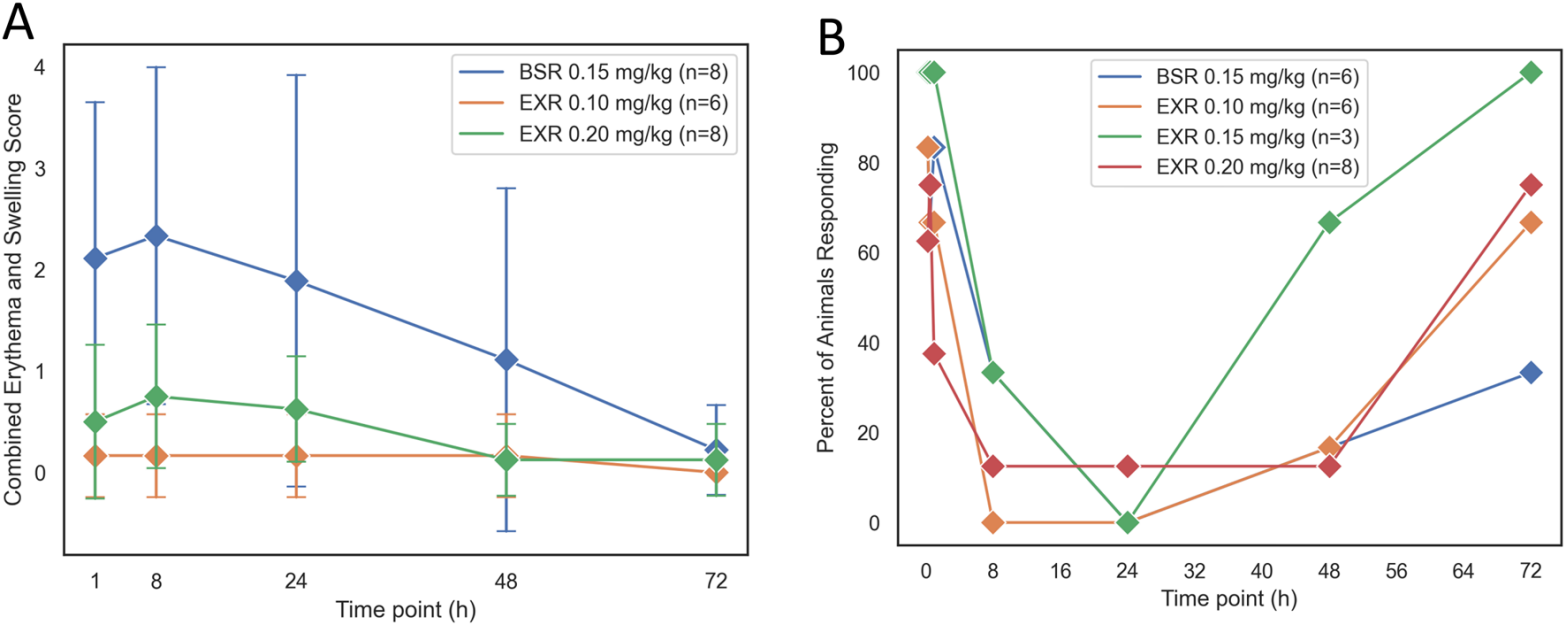
(**A**) Injection site scores based on erythema and swelling for animals in the PK cohort after a single injection of BSR or EXR. Data reported as mean ± standard deviation. (**B**) Percent of animals in PK cohort exhibiting reactivity (i.e., flinching, vocalizations, head turning) to needle insertion during phlebotomy events.

Throughout the PK study, all marmosets remained healthy. Mild sedation for BSR and all doses of EXR was observed at 8 and 24 h (data not shown). Moderate or severe sedation was not observed in any study animals. For PK study animals, injection site scores (based on erythema and swelling) were highest at 8 h after drug administration for both BSR and EXR (Fig. 2A). Injection site scores were highest in the BSR group, but were not statistically different from the other groups. Animals in the low dose EXR (0.1 mg/kg) had a minimal response. The injection sites in all PK study animals generally appeared normal by 72 h regardless of drug or dose. Normal activity was observed for all animals when returned to the home cage. Restraint, injection, and venipuncture did not result in any mobility changes in the study animals. Additionally, ataxia was not observed in any study animals. During phlebotomy, less than 20% of animals that received EXR at 0.1 to 0.2 mg/kg or BSR exhibited any reactivity (i.e., flinch, vocalization, head turn) to the needle insertion between 8 and 24 h (Fig. 2B).

All groups except EXR at 0.15 mg/kg had a statistically significant decrease in body weight at 24, 48 and 72 h with respect to baseline. However, these decreases were not clinically significant (< 10% of body weight). There was no significant difference between treatment groups (P-value > 0.05). One marmoset that received BSR lost > 10% of BW (10.33%) at 48 h after administration, but this normalized by 72 h.

### Video recordings

**Figure 3:**
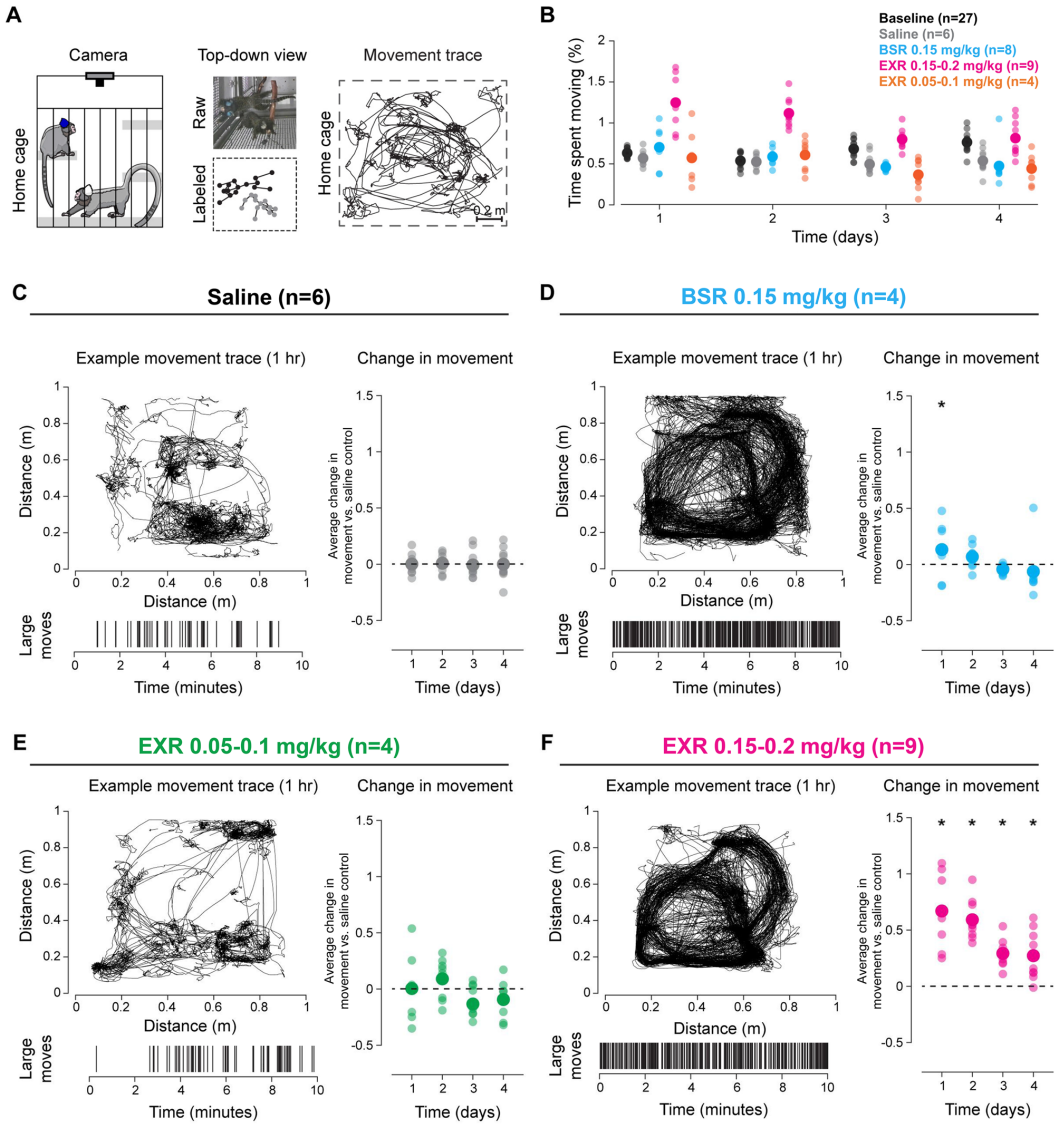
(**A**) Schematic of recording in home cage and movement tracing by machine learning algorithm. (**B**) Percent time spent moving between treatment groups for four consecutive days after administration of saline, BSR, or EXR. Baseline represents animal movement for four consecutive days before treatment. Large dots represent average, smaller dots represent individual animal per group. A representative movement trace (upper left), raster plot (lower left), and average change in movement compared to baseline (right) are shown for saline (**C**), BSR (**D**), EXR 0.05–0.01 mg/kg (**E**), and EXR 0.15–0.2 mg/kg (**F**). *P-value < 0.001.

Animal activity traces after drug administration were video recorded and quantified over time. For each treatment group, an example movement trace over 1 h is displayed in Fig. 3. Movement trace patterns were qualitatively more similar between saline and EXR at lower doses (0.05–0.1 mg/kg) compared to higher doses of EXR (0.15–0.2 mg/kg) (Fig. 3B). Saline injections did not cause any significant changes in activity levels on day 1 (day of dosing) or on days 2, 3 or 4 (Fig. 3C). A significant increase in activity was seen on day 1 in animals given BSR at 0.15 mg/kg (Fig. 3D). EXR at lower doses (0.05–0.1 mg/kg) did not cause any significant changes in activity levels (Fig. 3E). Higher doses of EXR (0.015–0.2 mg/kg) caused a significant increase in activity on days 1, 2, 3, and 4 (Fig. 3F).

### Histological analysis skin injection site after buprenorphine administration

**Figure 4:**
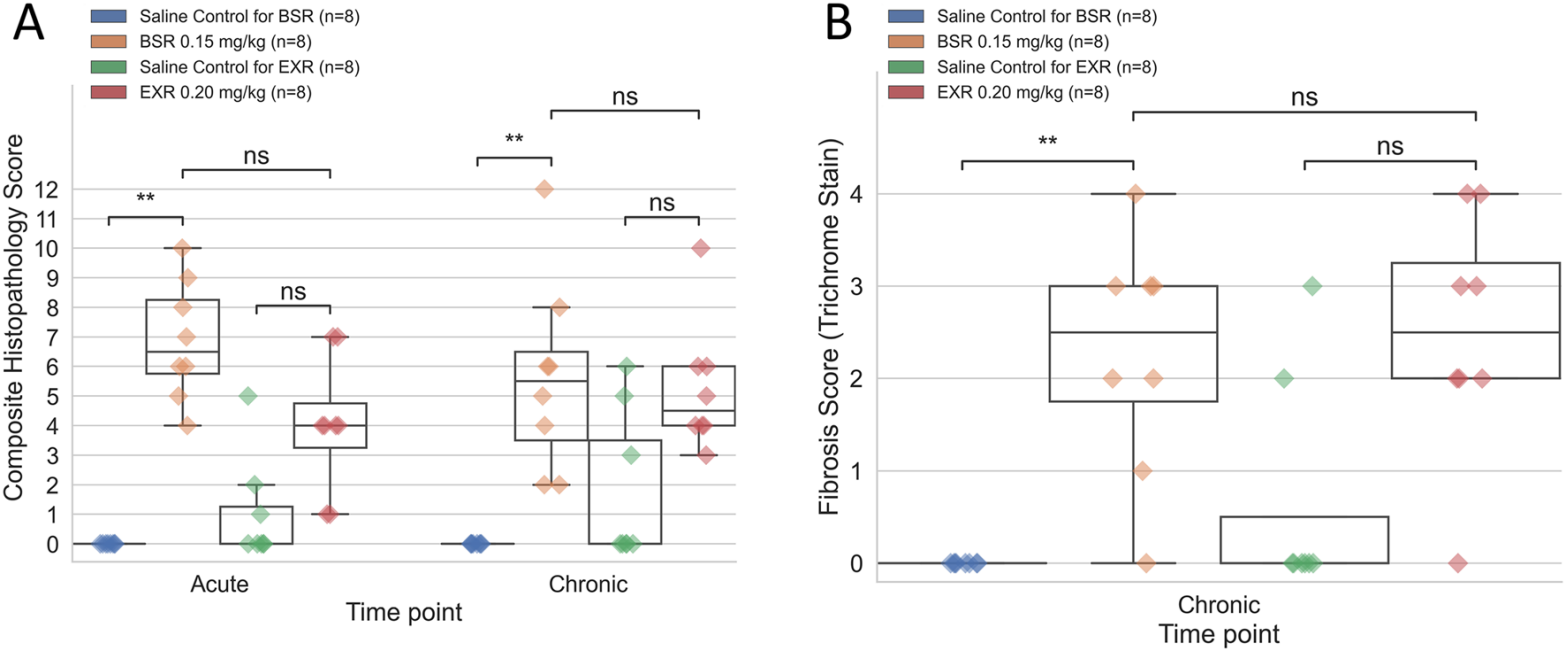
(**A**) Average composite histopathology scores for inflammation, necrosis, hemorrhage, edema, and fibrosis assessed by H&E stain of skin sections at injection site between BSR and EXR versus intra-animal saline controls at acute (24 h post-administration) and chronic (10 day post-administration) time points after drug administration. (**B**) Average fibrosis scores assessed by trichrome histological stain of skin sections at injection site between BSR and EXR versus intra-animal saline controls at chronic (10 day) time point after drug administration. **P-value < 0.01; ns, not significant.

**Figure 5:**
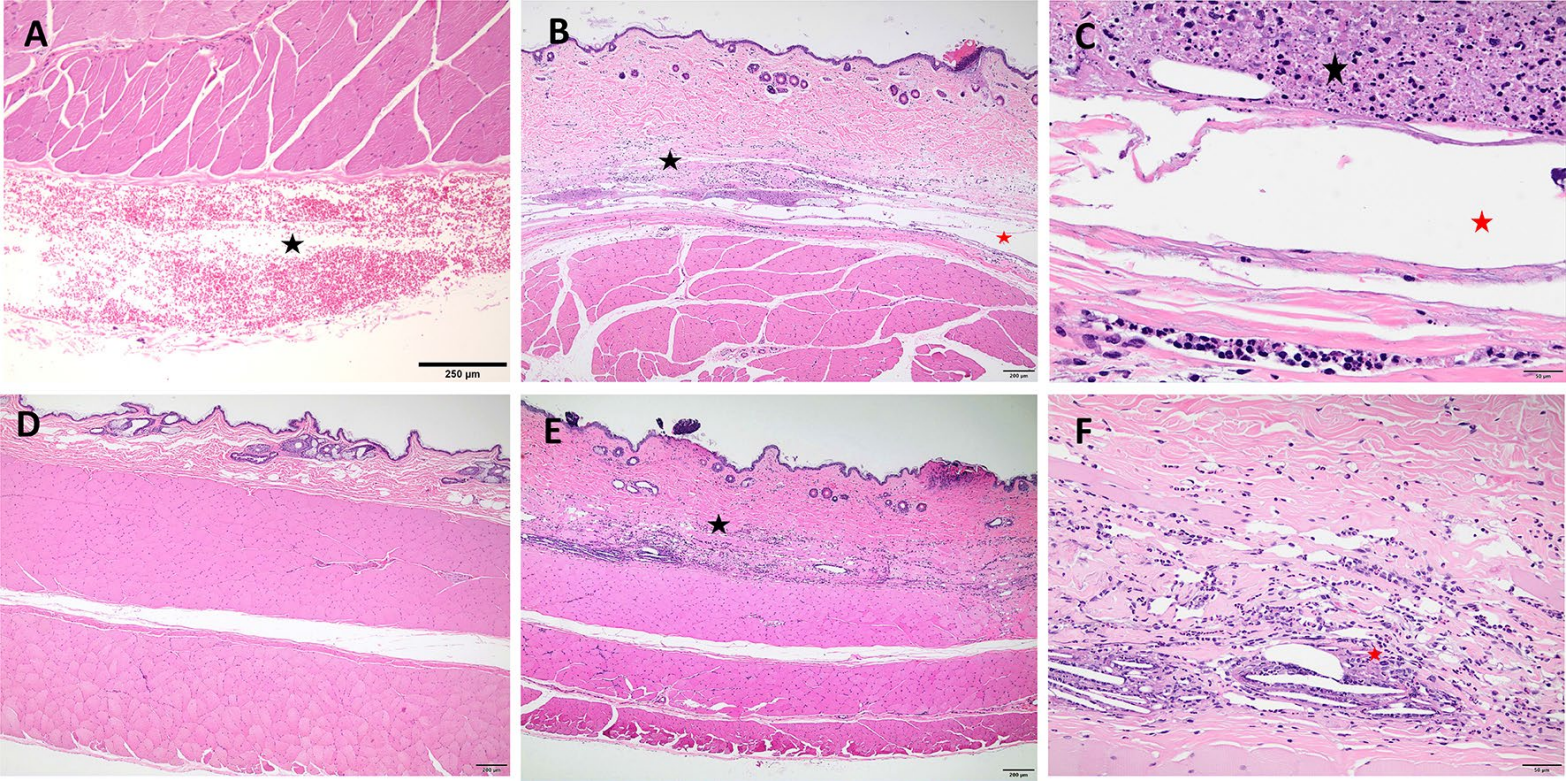
Representative H&E histology images of skin sections. Saline control site at acute time point (**A**) depicts acute hemorrhage (star) underneath deep musculature at the subcutaneous injection site (4 × magnification). BSR site at acute time point under 4 × magnification (**B**) depicts skin and underlying musculature with disruption of deep dermis (black star) and hypodermis by necrosis and inflammation (black arrows) with clear distended spaces comprising of injected material (red star). Focal epithelial thickening and hyperkeratosis is present at needle entry (injection) site (red arrow). BSR site at acute time point under 20 × magnification (**C**) depicts clear space associated with injected material (red star) surrounded by necrotic debris (black star) and degenerate neutrophils (black arrow). Saline control site at chronic time point (**D**) demonstrates normal skin and underlying abdominal musculature. EXR site at acute time point under 4 × magnification (**E**) of skin and underlying musculature shows disruption of deep dermis (black star) and hypodermis due to necrosis and inflammation (black arrows), with embedded clear needle-like crystals of injected material (yellow arrows). Focal epithelial erosion and necrosis is present at the injection site (red arrow). 20 × magnification of EXR site at acute time point (**F**) shows clear acicular crystals associated with injected material (black arrows) surrounded by degenerate neutrophils and necrotic debris (red star).

**Figure 6:**
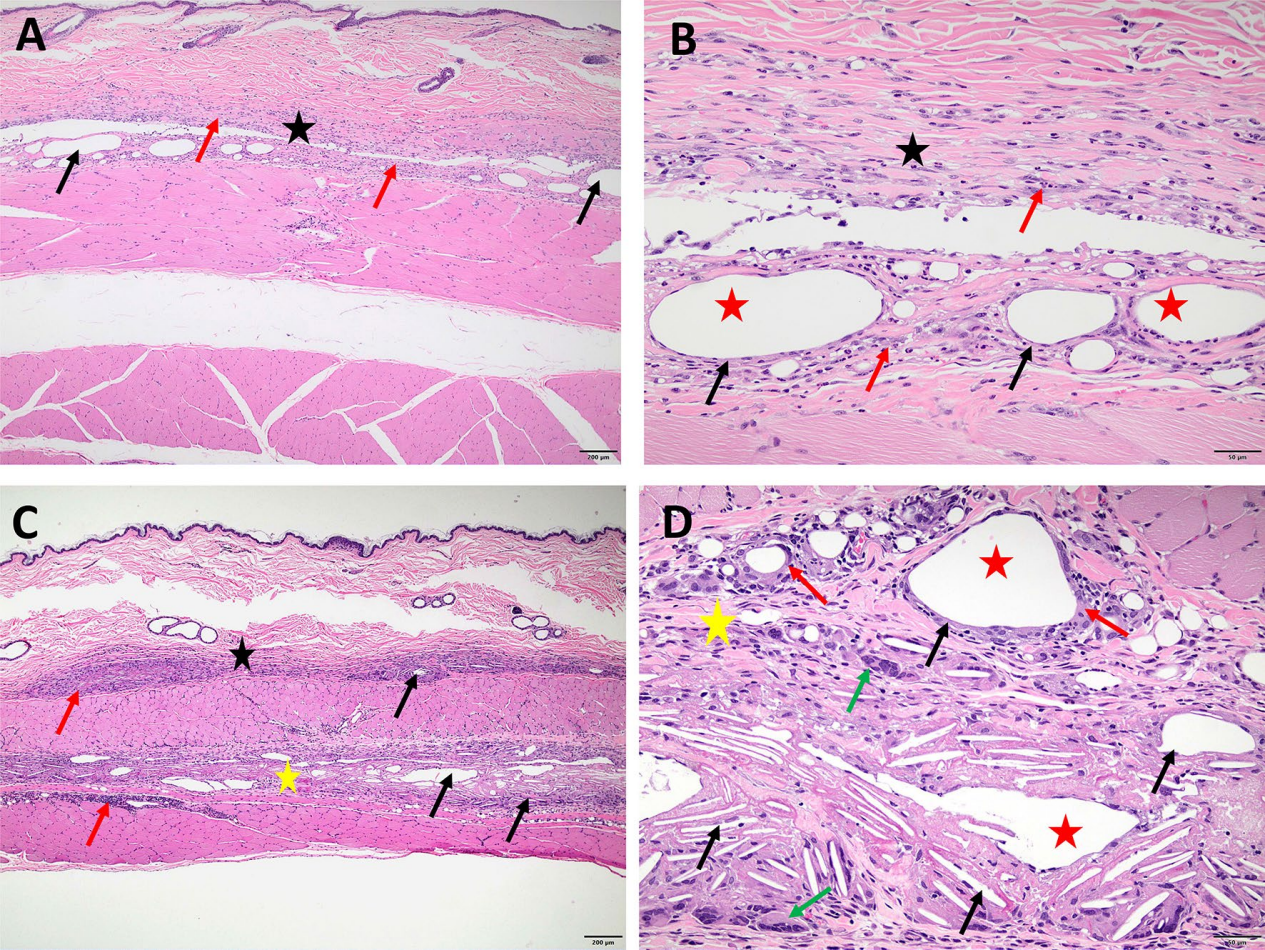
Representative H&E histology images of skin injection site sections. BSR site at chronic time point under 4 × (**A**) and 20 × (**B**) magnification depicts disruption of the deep dermis (black star) and hypodermis by necrosis/inflammation (red arrows) and multiple clear distended spaces (black arrows) associated with injected material (red star). EXR site at chronic time point under 4 × (**C**) and 20 × magnification (**D**) shows disruption of deep dermis/hypodermis (black star) and underlying musculature (yellow stars) due to necrosis/inflammation (red arrows), and multiple clear needle-shaped crystals (cholesterol clefts) (black arrows) and variably shaped distended spaces (yellow stars) are associated with injected material (red stars). An epithelioid cap (red arrows) surrounding injected material and multinucleated cells (green arrows) are also frequent (**D**).

At both acute and chronic time points, BSR but not EXR injection sites had significantly higher histopathology scores compared to saline controls (Fig. 4A). Both BSR and EXR acute injection sites exhibited acute necrosis and inflammation. The degree of inflammation was overall similar for chronic BSR and EXR injection sites but qualitatively different. BSR sites were associated with mainly macrophages and neutrophils, while EXR sites were associated with macrophages and multinucleated giant cells and frequently contained cholesterol clefts (due to the vehicle) (Figs. 5 and 6). Both drug formulations resulted in a fibroepithelial cap surrounding the injected material (Fig. 6 and Supplemental Fig. 1). Fibrosis scores based on trichrome stain evaluations were statistically higher for BSR compared to corresponding saline control sites (Fig. 4B). There were no statistically significant differences in fibrosis scores for EXR and corresponding saline control sites. Collagenolysis was absent for both treatment groups and controls (data not shown).

BSR resulted in an exaggerated inflammatory response in 1 out of 8 animals (12.5%). This was observed in an 11-year-old female marmoset that received BSR in the terminal skin biopsy cohort. At 72 h post injection, there was a round, raised, firm swelling approximately 1 cm in diameter, with erythema that extended beyond the circled region. This lesion was confirmed by histopathology to be a sterile pyogranuloma (Supplemental Fig. 1). The BSR from the same vial was cultured and negative for aerobic and anaerobic bacteria and fungal organisms. A fine-needle aspirate of the skin lesion at terminal collection was negative for bacterial growth. Gram, Periodic acid–Schiff (PAS), and Grocott methenamine silver (GMS) stains of histologic sections were negative.

### Buprenorphine and norbuprenorphine metabolism by liver microsomes

**Figure 7:**
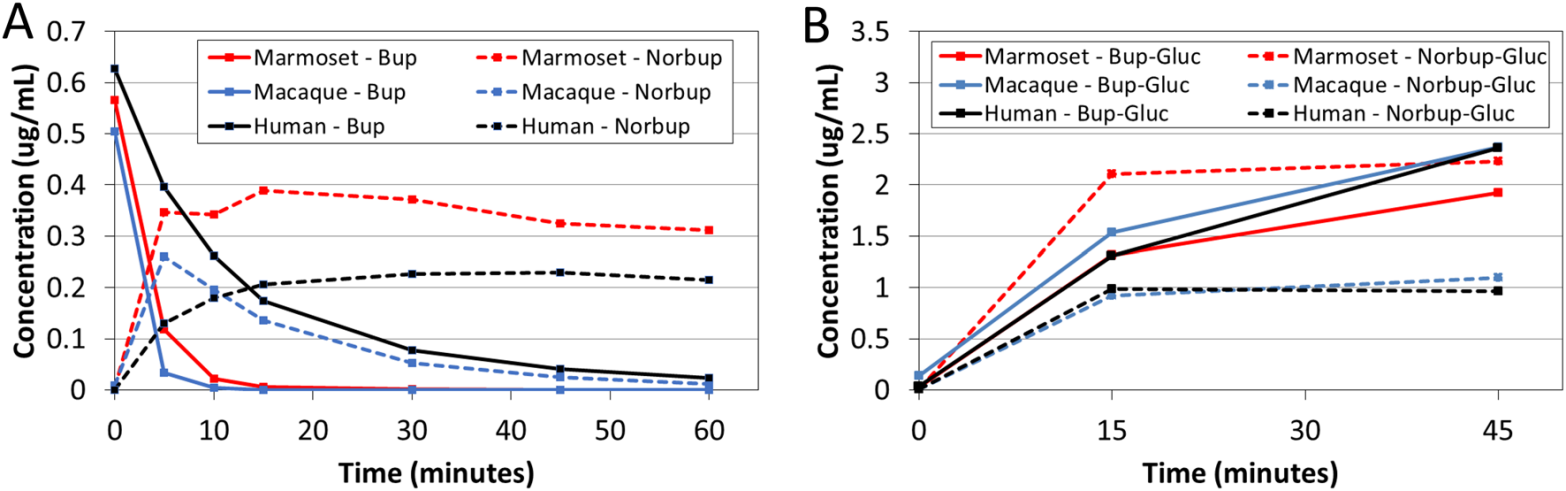
Buprenorphine and norbuprenorphine metabolism in vitro by marmoset, macaque, and human liver microsomes. (**A**) Cytochrome P450 conversion of buprenorphine (Bup) into norbuprenorphine (NorBup) after 60-min incubation. (**B**) Glucuronidation of buprenorphine or norbuprenorphine into buprenorphine-3-glucuronide (Bup-Gluc) and norbuprenorphine-3-glucuronide (Norbup-Gluc), respectively, after 45-min incubation. Data is for a representative experiment.

To compare the metabolism of buprenorphine by marmosets with other primate species, liver microsomes were used to study cytochrome P450 and glucuronidase dependent in vitro. Liver microsome from marmosets, macaques, and humans exhibited species-specific rates of buprenorphine conversion to norbuprenorphine via cytochrome P450 enzymes (Fig. 7A). By 15 min, both macaque and marmoset samples had metabolized all buprenorphine (Fig. 7A). In contrast, buprenorphine remained detectable at 60 min in samples with human liver microsomes. All three species generated norbuprenorphine from buprenorphine within 5 min (Fig. 7A). While macaques completely depleted norbuprenorphine by 45–60 min, the levels of norbuprenorphine plateaued by 5 min for humans and marmosets (Fig. 7A). Interestingly, by 60 min, marmosets produced appreciably higher concentrations of norbuprenorphine compared to humans and macaques (0.3 vs. 0.2 and 0 µg/mL, respectively) (Fig. 7A). In a separate experiment, glucuronidation of buprenorphine or norbuprenorphine was measured using liver microsomes (Fig. 7B). While marmosets exhibited comparable buprenorphine-3-glucuronide production as macaques and humans, marmoset liver microsomes produced higher amounts of norbuprenorphine-3-glucuronide at a more rapid rate (Fig. 7B).

## Discussion

Long-lasting analgesics are often preferred in NHP medicine and other species used in biomedical research, as less frequent dosing may result in reduced animal handling and stress and improved personnel compliance^14–16,20,32,33^. This study reports the pharmacokinetic profiles of BSR at 0.15 mg/kg and EXR at 0.1 mg/kg, 0.15 mg/kg and 0.2 m/kg in adult male and female common marmosets. Unlike BSR, EXR is a pharmaceutical grade, extended-release formulation of buprenorphine that is compliant with current Good Manufacturing Practices and FDA-indexed for mice and rats for post-procedural pain for up to 72 h^30^. This is the first study to evaluate EXR in marmosets, and we demonstrated that buprenorphine can be detected in the blood after EXR administration with a similar PK profile as BSR. There were no statistically significant differences in pharmacokinetic parameters such as elimination half-life, C_max_, and T_max_ between EXR and BSR. By evaluating multiple doses of EXR, from 0.1 to 0.2 mg/kg, we also demonstrated a dose-dependent response in mean plasma concentration–time curves. Importantly, EXR at 0.1 mg/kg, 0.15 mg/kg and 0.2 m/kg resulted in mean buprenorphine plasma concentrations that remained above the hypothesized therapeutic threshold of 0.1 ng/mL from the earliest measured time point of 15 min for at least 3 days.

At the Massachusetts Institute of Technology (MIT, Cambridge, MA), BSR is administered to marmosets at 0.15 mg/kg SQ. The mean C_max_ for the BSR group in this study was 1.82 ± 0.74 ng/mL. BSR at 0.15 mg/kg SQ in marmosets remained above the therapeutic threshold of 0.1 ng/mL until 129.5 h or 5.4 days after administration based on extrapolation of the terminal elimination phase of the mean concentration–time curve. However, some institutions may use a higher dose of BSR in marmosets. For example, Fitz et al. published the PK profile of BSR in marmosets dosed at 0.2 mg/kg SQ, resulting in a mean C_max_ of 2.78 ± 1.19 ng/mL. BSR at 0.2 mg/kg remained above the therapeutic threshold of 0.1 ng/mL until 92.7 h after administration, and a dosing frequency of 3–3.5 d was recommended^14^. Minimal ataxia and mild sedation peaked at 8 h after administration of BSR at 0.2 mg/kg^14^. Similarly, in our study, animal sedation peaked at 8 h after administration of BSR at 0.15 mg/kg. Additionally, no differences in sedation were observed between BSR and EXR, and ataxia was not noted in any group.

In addition to direct and cage-side clinical evaluations of study animals, video analysis was performed in a different cohort of animals to indirectly observe and quantify activity levels after drug administration over longer periods of time (up to 4 days after dosing). Since buprenorphine is known to cause ataxia and sedation, we used a machine learning algorithm capable of detecting activity changes that could be associated with these adverse effects. Video analysis found that BSR and EXR at higher doses altered animal typical movement patterns within the home cage when compared with saline control. Interestingly, animals exhibited increased locomotor activity following BSR at 0.15 mg/kg and EXR at 0.15 mg/kg and 0.2 mg/kg doses (higher doses), but not at EXR at 0.05 mg/kg and 0.1 mg/kg (lower doses), compared to saline-treated controls. Activity levels between EXR at 0.05 mg/kg and 0.1 mg/kg and saline-treated animals were not significantly different. These results show that buprenorphine is associated with dose-dependent hyperactivity in marmosets. Ataxia and sedation were not observed in the video recordings. This paradoxical increase in locomotor activity possibly represents disinhibition of activity circuits, which has been described in multiple animal species receiving opioids, including rats, mice and horses^34–38^. While this increase in activity may not cause clinical concerns in most cases, researchers studying behavior and neurobiology should be aware of and take into account this potential effect in marmosets and possibly other NHP species when analyzing study data. Additionally, increased activity may be of concern in certain clinical circumstances, such as animals that are in the acute recovery period after cranial implantation or other invasive surgeries.

Our study did not evaluate the pharmacodynamics of buprenorphine, as no published or validated methods are available to assess pain or analgesic efficacy in marmosets^5^. Additionally, buprenorphine can significantly impact the behavior of normal (unoperated) animals such as rats, making assessments of efficacy challenging with traditional systems used to score clinical pain^39,40^. Instead, we considered the therapeutic threshold to be 0.1 ng/mL based on buprenorphine efficacy studies in dogs and humans, which has also been used as a target in macaques and marmoset buprenorphine PK studies^12,14–16,20^.

Clinical signs of respiratory depression were not observed in any study animal administered BSR or EXR. However, marmosets have been reported to be more susceptible to respiratory depression after being administered buprenorphine doses that are considered safe in macaques, rodents, and other laboratory animal species^14,16,17,19,41^. Respiratory depression by buprenorphine has been attributed to its major metabolites, norbuprenorphine and norbuprenorphine-3-glucuronide, produced by cytochrome P450 (CYP) and UDP-glucuronosyl transferase (UGT) enzymes, respectively^10,42,43^. Simultaneous detection of both buprenorphine and norbuprenorphine has been described previously in macaques^16^. However, this method historically required large plasma sample volumes (~ 1 mL), and due to the blood volumes constraints for marmosets, previous investigations of buprenorphine pharmacokinetics in marmosets have been limited to studying the parent drug^16^. In our study, we optimized an LC–MS/MS method for detection of both buprenorphine and norbuprenorphine in small plasma volumes (100 µL sample aliquot) to accommodate the limited blood volume available from marmosets. Using this improved LC–MS/MS method, norbuprenorphine was detected in marmoset plasma after administration with BSR or EXR. While we observed considerable inter-individual variation in the norbuprenorphine concentrations, our results indicate marmosets produce this metabolite after BSR or EXR treatment. Future investigation is required to determine if age, sex, hepatic function, or other factors influence norbuprenorphine accumulation in marmosets and are risk factors for respiratory depression or other adverse side effects. In humans, sex-related differences exist in the pharmacokinetics of buprenorphine in the plasma^44^. Specifically, women have significantly higher area under the plasma concentration curve (AUC) and maximum plasma concentrations for buprenorphine, norbuprenorphine and norbuprenorphine-3-glucuronide. In contrast, lower plasma buprenorphine concentrations have been noted in female versus male rats after treatment with EXR^30^. A limitation of our study was that it was not powered to identify if sex, age, or other factors affect the PK profiles of buprenorphine or its metabolites.

We also validated that marmosets produce norbuprenorphine and glucuronide conjugates in vitro using liver microsomes assays of CYP- and UGT-mediated metabolism. The liver microsome results from this study suggest that marmosets and macaques both metabolize buprenorphine rapidly, but that marmosets metabolize norbuprenorphine at a slower rate than macaques. Interestingly, liver microsomes from marmosets produced norbuprenorphine and glucuronide conjugate more rapidly compared to humans and macaques, and these metabolite profiles were comparatively sustained in marmosets. These in vitro findings correspond to clinical observations of increased susceptibility for respiratory depression in marmosets. Homologous CYPs and UGTs genes have been described in marmosets^45,46^. However, additional studies are required to further understand species-specific differences in the expression and/or activity of CYP- and UGT-mediated metabolism of buprenorphine in marmosets. The microsome data suggest that marmosets may have rapid production of and delayed clearance of norbuprenorphine, leading to sustained exposure to norbuprenorphine. A caveat of our in vitro metabolism experiments was the limited availability of marmoset liver samples to prepare microsomes, which permitted only a representative experiment evaluating CYP- and UGT-mediated metabolism. Future studies may be needed to evaluate plasma norbuprenorphine concentrations beyond 72 h after extended-release buprenorphine administration in marmosets as well as to determine if this increases risks for respiratory depression.

BSR is not pharmaceutical grade and thus its use in research animals may not be compliant with federal regulations. Federal regulations require that compounds used in research animals be pharmaceutical grade. According to the 8th edition of the *Guide*^8^, the use of pharmaceutical-grade chemicals and other substances ensures that toxic or unwanted side effects are not introduced into studies conducted with experimental animals. EXR is a pharmaceutical grade formulation FDA-indexed for mice and rats. As a result of it being pharmaceutical grade, EXR is compliant with the *Guide*.

Other long-lasting buprenorphine formulations are available for use in veterinary species. Simbadol (Zoetis) is a long-lasting, highly concentrated buprenorphine solution (1.8 mg/mL) that is FDA-approved for postoperative pain in cats SQ once daily for up to 3 days. In macaques, Simbadol appeared to provide longer plasma concentration durations compared to the regular buprenorphine HCl formulation^15^. However, the use of Simbadol in marmosets as a long-lasting analgesic opioid is less practical because the drug is too concentrated to allow accurate dosing in this small primate species (e.g., 300–450 g)^4^ and cannot be diluted. Additionally, Simbadol has been associated with seizures in rabbits^9^, suggesting some species may experience severe adverse events. Zorbium (Elanco) is a recently FDA-approved long-acting transdermal buprenorphine formulation to control postoperative pain in cats for 4 days. Future studies investigating the use of Zorbium in marmosets could be performed with the caveat that this product requires a minimum drying time of 30 min after application, which may be more time consuming than other analgesic delivery methods, or could be compromised by allogrooming if improperly applied. Butrans (Purdue Pharma), a buprenorphine transdermal patch (TDP) FDA-approved for humans, has been studied in cynomolgus macaques acclimated to wearing primate jackets^47^. The 10- and 20-µg/h TDP requires approximately 12–24 h to reach the hypothesized therapeutic threshold of 0.1 ng/mL and remains above this threshold for 5 to 6 days post-placement, respectively. Additionally, other analgesics are recommended during the immediate postoperative period or placement of the TDP 24 h preoperatively due to the delayed absorption. These considerations make the use of TDP products in marmosets less practical compared injectable long-lasting buprenorphine formulations.

BSR has been reported to cause injection site reactions in multiple species, including macaques^16,22^, mice^23,24^, rats^21,48^, guinea pigs^25^, prairie dogs^26^, elephant seals^27^, dogs^28^, and swine^29^. BSR at 0.2 mg/kg was also reported to result in mild localized erythema and skin swelling in 1 out of 6 marmosets^14^. Despite routine use of BSR in laboratory marmosets, the prevalence of injection site reactions associated with this formulation has not been reported in the literature. In general, BSR injection site skin reactions have been attributed to the proprietary drug vehicle^22,24^, a biodegradable polymer of dl-lactide-co-caprolactone copolymers [poly(DLLA-co-CL)] with N-methyl-2-pyrrolidone (NMP) as a solvent. This matrix is hydrophobic, water-insoluble, and precipitates in body fluids to form a gel depot for the release of buprenorphine. The copolymer poly(DLLA-co-CL) has been reported to remain subcutaneously for up to 2 years in rats, where it elicits a foreign body reaction resulting in the eventual degradation of the polymers by multinucleated giant cells^49,50^. In rhesus macaques, injection site reactions related to 3 mg/mL BSR have been noted as long as 311 days after administration, and risk factors for this species included the MHC allele Mamu-B*29 and body weight, i.e., a dose-dependent exposure to the copolymer^22^. In contrast, EXR is encapsulated within solid lipid nanoparticles (SLN) and suspended in a medium chain fatty acid triglyceride, which is degraded over time via lipase and esterase activity^38,51^.

Refinement of drug delivery should always be considered for animal comfort and welfare. For this study, we aimed to use the smallest needle size possible for the administration of each formulation to elicit the least amount of pain in study animals during injection. We determined that EXR can be administered using smaller needle sizes due to its lower viscosity compared to BSR (28-gauge needle for EXR, allowing the use of insulin syringes, versus 22-gauge for BSR). We observed that the administration of EXR was consistently associated with less bleeding and drug leakage at the injection site compared to BSR. Difficulty administering BSR has been described in the literature in mice due to the viscosity, which requires larger needle sizes^24^. We hypothesize that vehicle leakage on the skin may contribute to gross signs of injection site reactions.

To histopathologically evaluate injection site skin reactions caused by BSR and EXR in marmosets, skin biopsies were collected 1 day (acute) and 10 days (chronic) post-drug administration at necropsy and scored for inflammation, necrosis, hemorrhage, edema, and fibrosis. BSR injection sites exhibited significantly elevated histopathological scores compared to saline control sites than for EXR. This finding suggests EXR may cause less severe injection site reactions compared to BSR, which we hypothesize is attributed to the differences in vehicle. However, as the vehicle is proprietary for both formulations, we were not able to evaluate the effects of BSR and EXR vehicles without drug in our study. Both EXR and BSR injection sites exhibited fibrosis, but fibrosis was significantly elevated for BSR. Our findings suggest that repeated injections of these drugs at the same anatomical site, especially for BSR, should be avoided due to the potential for fibrosis. Our histopathological evaluations showed that BSR injection sites were associated with mainly macrophages and neutrophils. In contrast, EXR sites were associated with macrophages and multinucleated giant cells, which is consistent with what has been previously described in rats administered EXR (0.65 to 1.3 mg/kg SQ)^30^.

A limitation of our study was that semi-quantitative clinical assessment injection site reactions were not feasible in the histopathology cohort because stress associated with daily home cage removal and restraint to measure erythema and palpate for swelling was not justified. Cage-side visual inspections of animals were performed but were not practical to provide quantitative evaluations. To overcome this limitation, animals in the PK cohort were clinically assessed for injection site reactions since removal from the home cage and restraint was necessary during blood collection time points. While there were no statistical differences swelling and erythema scores, animals receiving BSR has higher average scores at 1–48 h compared to EXR.

In conclusion, we recommend the use of EXR at 0.1 to 0.15 mg/kg as an alternative to BSR for longer-lasting analgesia in marmosets because both formulations exhibit comparable rapid absorption and remain above the therapeutic threshold for at least three days post-administration. Additionally, EXR is superior to BSR because the lower viscosity of this formulation lends to greater ease and safer of administration. We also observed less intense injection site reactions with EXR compared to BSR, suggesting a lower risk for adverse events. Most importantly, we recommend EXR as an alternative to BSR as a long-lasting opioid analgesic in marmosets because unlike BSR, EXR is pharmaceutical-grade and thus compliant with the *Guide for the Care and Use of Laboratory Animals* and the Animal Welfare Act and Regulations.

## Methods

### Animals

Adult marmosets in the PK study were divided into four groups: BSR 0.15 mg/kg group (1.4–3.4 years old; 4 males, 4 females), and EXR 0.1 mg/kg group (2.0–6.2 years old; 3 males, 3 females), EXR 0.15 mg/kg group (2.5–5.9 years old, 0 males, 3 females), and EXR 0.2 mg/kg group (1.9–6.1 years old; 4 males, 4 females). Adult marmosets were included in the video recording study, which were divided into five groups: saline group (1.7–3.8 years old; 3 males, 3 females), BSR 0.15 mg/kg group (3.0–6.1 years old; 2 males, 2 females), EXR 0.05 mg/kg group (3.5 years old; 0 males, 1 females), EXR 0.1 mg/kg group (2.3–2.8 years old; 2 males, 1 females), EXR 0.15 mg/kg group, (2.5–5.9 years old; 0 males, 3 females), and EXR 0.2 mg/kg group, (1.7–3.4 years old; 3 males, 3 females). Adult marmosets scheduled for unrelated terminal procedures were included in a separate cohort to evaluate drug injection site skin histopathology. These animals were divided into two groups: BSR 0.15 mg/kg group (2.3–12 years old; 4 males, 4 females) and EXR 0.2 mg/kg group, (3.0–10.5; 4 males, 4 females).

All study procedures were performed under approval from the MIT Committee on Animal Care and followed all applicable federal, state, and local guidelines and regulations. All marmosets were housed and maintained in an AAALAC-accredited facility according to the standards in the Animal Welfare Act and Regulations, Public Health Service Policy, and the *Guide for the Care and Use of Laboratory Animals*^6,8,52^. Methods were carried out in accordance with the ARRIVE guidelines. Animals were not fasted or sedated for any drug administration or blood collection events.

Subject selection criteria for the PK and video recording studies were as follows: animals were considered clinically healthy, with a body condition score (BCS) of at least 3 on a scale of 1 to 5^53,54^. All animals were > 300 g in body weight, not pregnant or nursing, not on any medications or receiving any other research manipulations at the time of the study. All marmosets were socially housed (mixed or same-sex adult pairs or in a family group) in enriched Britz cages (30 in. × 32 in. × 67 in.). Enclosures contained perches, nest boxes, hammocks, manzanita wood branches, and hanging toys. Housing rooms were maintained at 78.0 ± 2.0°F (23.3 ± 1.0 °C) with a relative humidity of 30% to 70%. Full spectrum lighting was provided on a 12:12-h light:dark cycle. The diet consisted of extruded biscuits (Teklad New World Primate Diet 8794, Envigo, Madison, WI, and LabDiet New World Primate 5040, Purina, St. Louis, MO) soaked lightly in water, supplemented with canned diet (ZuPreem, Premium Nutritional Products, Shawnee, KS), gel diet (Mazuri Callitrichid Gel Diet #5B34), washed fruits and vegetables, and various protein sources such as eggs, yogurt, cottage cheese, and beans. Enrichment included acacia gum (Bio-Serv, NF/FCC, F7015), foraging trays, and mealworms. Chlorinated reverse-osmosis-purified water was provided ad libitum*.*

Prior to inclusion in the PK and video recording studies, animals were determined to be healthy based on complete physical, hematological, and biochemical examinations performed semiannually. All animals were seronegative for squirrel monkey cytomegalovirus, *Saimiriine herpesvirus 1*, *Saimiriine herpesvirus 2*, and measles virus on arrival to the facility (C panel, VRL Laboratories, San Antonio, TX). Veterinary staff observed marmosets at least twice daily, and individual health records were maintained.

### Drugs

Marmosets received either Buprenorphine SR-LAB (BSR) (1 mg/mL, ZooPharm, currently renamed as Buprenorphine ER-LAB) at 0.15 mg/kg SQ using a 1-mL low dead-space syringe (22 g, ¾ inch needle) or Ethiqa XR (EXR) (1.3 mg/mL, Fidelis Animal Health) at 0.1 mg/kg, 0.15 mg/kg, or 0.2 mg/kg SQ using a ½-mL tuberculin syringe (28 g, ½ inch needle). The drug was administered at the ventral abdomen for ease of administration and monitoring injection sites. All injection sites were circled with a surgical skin marker immediately after administration to facilitate monitoring during the observation period as well as tissue collection. For all drug dosing events, one person manually restrained the animal while a second person administered the drug. Care was taken so that the drug was not injected directly into the circulation (intravenously).

### Blood sample collection

Blood samples (approximately 0.25–0.3 mL each) were collected from animals under manual restraint from the saphenous vein using a 27-gauge, thin-walled needle and 1-mL low dead-space syringe. For all blood sample collections, one person manually restrained the animal while a second person performed the phlebotomy. Samples were immediately transferred from the syringe into heparinized tubes. Plasma was then immediately isolated by centrifugation at 2000×*g* for 10 min and stored at − 80 °C until shipment to the Center of Human Toxicology, at the University of Utah. Time points 15 min, 30 min, 1, 8, 24, 48 and 72 h were collected. Over the course of the entire study, the blood volume removed from each marmoset was calculated to be < 10% of the animal’s total blood volume.

### Animal observations

Marmosets were directly observed by veterinary staff for adverse effects such as lethargy, sedation, respiratory depression, changes in appetite, nausea, and diarrhea for up to 2 h after drug administration. Animals were also monitored closely for local and general adverse effects due to drug injection and blood sampling such as bruising, hematoma formation, and changes in mobility. Ataxia and sedation were rated at each time point by the same observer (NF) using a numeric scoring system. Scores (0, none; 1, mild; 2, moderate; and 3, severe) were assigned according to inclusion criteria (Supplementary Tables 1 and 2). The injection site was also rated at each time point using a numeric scoring system by the same observer (NF). Scores (0, none; 1, minimal; 2, mild; 3, moderate; 4, moderate to severe; 5, severe) were assigned according to inclusion criteria (Supplementary Table 3). Needle reactivity during each phlebotomy time point were scored by the same observer (NF) using a binary scoring system (Supplementary Table 4).

The health of the marmosets was monitored throughout the course of the study. Marmosets were evaluated at least twice a day by veterinary and animal care staff by cage-side observation for injection site reactions and changes in behavior, activity level, and fecal output. Body weight was measured each time a marmoset was manipulated during the study.

### Video recordings

In-cage activity measurements of individual marmosets housed in pairs (separate cohort from PK study animals) were made using a non-invasive video camera set up for long-term recordings. Recordings were analyzed using machine learning algorithms based on previously published methods using DeepLabCut^55^. Ear tufts were colored with an animal-safe dye (Stoelting Co, Wood Dale, IL) to distinguish cage mates. Both marmosets in each recorded cage were given BSR, EXR, or saline. Four days of baseline recordings (pre-injection) were performed for each animal to allow for acclimation to the camera set up. Animals were recorded after they were returned to the home cage within 30 min after injection and for 3 additional days after injection during the lights-on period (between 7:00 am and 7:00 pm). Recordings of animals were performed for the following groups: BSR at 0.15 mg/kg SQ (n = 4), EXR at 0.05 mg/kg (n = 1), EXR at 0.1 mg/kg (n = 3), EXR at 0.15 mg/kg (n = 3), EXR at 0.2 mg/kg (n = 6), and saline control (n = 6). All injections were done at the same time of day (between 8:00 a.m. and 10:00 am). Results from EXR at 0.05 EXR at 0.1 mg/kg (n = 3) were not statistically different and were thus combined into one group (n = 4). Results from EXR at 0.15 EXR (n = 3) at 0.2 mg/kg (n = 6) were also not statistically different and were combined into one group (n = 9).

### Plasma and microsomal drug/metabolite analysis

Plasma and microsomal concentrations of buprenorphine, norbuprenorphine, and their glucuronides were measured using assays adapted from an existing, validated liquid chromatography–electrospray ionization–tandem mass spectrometry (LC–ESI–MS/MS) method previously used in both human and veterinary studies^16,56^. For both sample types, a Thermo Scientific Vanquish autosampler and LC pump was interfaced with a Thermo Scientific Velos Pro linear ion trap MS (San Jose, CA) operated in the Selected Reaction Monitoring (SRM) mode under positive ionization conditions. All reference materials were obtained from Cerilliant (Round Rock, TX).

Following addition of the internal standards (buprenorphine-d_4_ and norbuprenorphine-d_3_), a 100 µL aliquot of each plasma sample was extracted using liquid–liquid extraction under basic conditions. Briefly, 10% ammonium hydroxide solution was added to each sample, followed by the addition of methyl tert-butyl ether (MTBE) to extract buprenorphine, norbuprenorphine, and their internal standards from plasma. After freezing, the upper MTBE layer was decanted into a fresh tube, dried, and the sample residue was reconstituted in 50 µL of 5:95, acetonitrile:0.1% formic acid (aqueous). A 30 µL aliquot of the reconstituted sample was injected for analysis. Chromatographic separation was performed on a Waters (Milford, MA) Acquity® BEH C18 column (2.1 × 50 mm, 1.7 µm particle size) with gradient elution using 0.1% formic acid (aqueous, A) and acetonitrile (B) as mobile phases. Initial mobile phase conditions were held for 0.5 min at 5% B, then linearly increased to 35% B until 5 min, held at 35% B until 6.5 min, prior to rapidly returning to 5% B at 6.6 min. to re-equilibrate the column for 4.4 min. For buprenorphine, buprenorphine-d_4,_ norbuprenorphine, and norbuprenorphine-d_3,_ the mass/charge (m/z) SRM transitions (collision energy, CE) were 468.3 to 414.2 (35%), 472.3 to 415.2 (35%), 414.2 to 396.2 (30%), and 417.2 to 399.2 (30%), respectively. The lower limit of quantitation (LLOQ) was 0.1 ng/mL for both analytes.

For microsome incubation samples, a 25 µL aliquot was diluted with 400 µL of 5:95, acetonitrile:0.1% formic acid (aqueous) following addition of the internal standards (buprenorphine-d_4_, norbuprenorphine-d_3_, buprenorphine glucuronide-d_4_, and norbuprenorphine glucuronide-d_3_). A 30 µL aliquot of the reconstituted sample was injected. Chromatographic separation was performed on a Phenomenex (Torrance, CA) Synergi™ Polar-RP column (2.0 × 100 mm, 2.5 µm particle size) with the same gradient elution and mobile phases as the plasma method, above. The same SRM transitions and collision energies as the plasma method were used for buprenorphine, buprenorphine-d_4,_ norbuprenorphine, and norbuprenorphine-d_3_. SRM transitions (CE) for buprenorphine glucuronide, buprenorphine glucuronide-d_4_, norbuprenorphine glucuronide, and norbuprenorphine glucuronide-d_3_ were 644.3 to 468.3 (20%), 648.3 to 472.3 (20%), 590.3 to 414.2 (25%), and 593.3 to 417.2 (25%), respectively. The lower limit of quantitation (LLOQ) for the microsome samples was 10 ng/mL for all analytes.

### Pharmacokinetic data analysis of plasma samples

Results from each marmoset were grouped according to drug formulation, dose, and time point. The mean plasma concentration of buprenorphine was plotted for the time points to generate plasma concentration–time curves, which were compared between BSR and EXR groups. The mean plasma concentration of the metabolite norbuprenorphine was also plotted to generate plasma concentration–time curves for BSR and EXR groups. Pharmacokinetic parameters were calculated using a noncompartmental analysis for extravascular administration using Phoenix WinNonLin version 8.1 (Certara, Princeton, NJ). Calculated parameters, including maximum observed plasma concentration (C_max_), time to maximum observed plasma concentration (T_max_), observed time of last quantifiable plasma buprenorphine concentration (T_last_), terminal elimination half-life (t_1/2_), area under the curve from time 0 extrapolated to T_last_ (AUC_0–Tlast_), area under the curve from time 0 extrapolated to infinity (AUC_0–∞_), clearance, the elimination rate constant (k_e_), observed mean residence time (MRT), and observed mean residence time to infinity (MRT_0–∞_), were determined from the individual observed concentration–time data. AUC_0–Tlast_ was obtained by using the linear up–log down trapezoidal rule. The area was extrapolated to infinity (AUC_0–∞_) using the rate constant of the terminal k_e_. The terminal half-life (t_1/2_) was calculated by dividing the natural logarithm of 2 by k_e_. Clearance (outputted as Cl_F_obs by Phoenix WinNonLin) represents total body clearance for extravascular administration calculated by dividing dose over AUC_0–∞_. A target plasma concentration of 0.1 ng/mL was used in this study, which has been reported to be efficacious in humans and dogs, and was the target concentration used in previous buprenorphine pharmacokinetic studies in marmosets and macaques^14–16,19,20,57^.

### Microsome extraction from marmoset liver samples

Marmoset liver was collected from adult animals (2 males, 5 females) at necropsy and stored in 1.15% KCl buffer at − 80 °C. Microsomes were prepared using the Microsome isolation kit (Abcam, ab206995) following the manufacturer’s instructions and pooled at equal total protein concentrations.

### Buprenorphine and norbuprenorphine metabolism by liver microsomes

250 µg total protein of pooled liver microsome from marmosets, Cynomolgus monkey (ThermoFisher, MKMCPL), or human (ThermoFisher, HMMCPL) microsomes were incubated with 10 µM of buprenorphine (Sigma) or 10 µM norbuprenorphine (Cayman Chemicals) and RapidStart NADPH Regenerating System (5 mM glucose 6-phosphate, 1 mM NADP, 1 unit of glucose-6-phosphate dehydrogenase, XenoTech, K5000) in incubation buffer (0.1 M phosphate buffer, 1.0 mM EDTA pH 7.4, 5.0 mM MgCl_2_) at final volume of 500 µL. Reactions were incubated for 3 min at 37 °C before adding 50 µL of the NADPH regenerating system to initiate metabolism. At 0, 5, 10, 15, 30, 45, and 60 min, 50 µL of reaction volume was removed and added to 100 µL of ice-cold 50% methanol. Samples were incubated on ice 15 min and then centrifuged at 20,000 xg for 10 min at 4 °C. Supernatant was collected and stored at − 80 °C. For glucuronidation reactions, 100 µg total protein of pooled liver microsome from marmosets, Cynomolgus monkeys, or humans were incubated with 10 µM of buprenorphine (Sigma) or 10 µM norbuprenorphine (Cayman Chemicals) with 25 µg/mL alamethicin (Cayman Chemicals), 5 mM saccharolactone (Cayman Chemicals), and 2 mM UDPGA (Cayman Chemicals) in incubation buffer (0.1 M phosphate buffer, 1.0 mM EDTA pH 7.4, 5.0 mM MgCl_2_) at final volume of 200 µL. Reactions were incubated for 3 min at 37 °C before adding UDPGA to initiate metabolism. At 0, 15, and 45 min, reactions were terminated by adding 100 µL of ice-cold methanol to 50% volume/volume. Samples were incubated on ice 15 min and then centrifuged at 20,000×*g* for 10 min at 4 °C. Supernatant was collected and stored at − 80 °C until shipment to University of Utah Center for Human Toxicology for metabolite detection, as described above.

### Necropsy and skin biopsy collection

Marmosets received either BSR at 0.15 mg/kg (n = 8) or EXR at 0.2 mg/kg SQ (n = 8). Each animal received a single chronic dose (approximately 10 days before euthanasia) and acute dose (1 day before euthanasia) of either drug along with a volume-matched sterile saline (0.9%, USP grade) control injection at separate sites on the ventral abdomen. Thus, each animal had a total of 4 separate injection sites at the time of euthanasia and skin biopsy. All injection sites were circled with a surgical skin marker immediately after drug administration. Just prior to skin biopsy collection, animals were sedated with a combination of alfaxalone and ketamine given intramuscularly in the quadriceps muscle, and deeply anesthetized with intravenous pentobarbital sodium euthanasia solution at 30 mg/kg (FATAL-PLUS solution, Vortech Pharmaceuticals, Dearborn, MI, diluted to 50 mg/mL). Phosphate buffered saline (PBS) was used for perfusion to remove the circular blood, and then perfusion with 4% paraformaldehyde in PBS was performed to fix the tissues. Full thickness skin biopsies approximately 2 cm × 2 cm in size and containing the injection site in the center were excised with a scalpel and placed in 10% formalin. Each skin site was placed in its own formalin-containing specimen cup and labeled with a randomly generated identification code to ensure that the pathologist who evaluated the samples was blind to the treatment group.

### Tissue processing and histopathology scoring

Formalin-fixed skin samples were cut in parallel sections to generate approximately 6 tissue sections from each sample. Sections were embedded in paraffin, cut in 5-µm sections, and stained with hematoxylin and eosin. Sections were examined by a board-certified veterinary pathologist (SM) and graded semi-quantitatively for inflammation, hemorrhage, edema, necrosis, epidermal hyperplasia, and mineralization. Skin sections of the chronic drug and saline control injection sites were also stained with Masson trichrome and scored for fibrosis and collagenolysis. Each criterion was scored on a scale from 0 to 5 as follows: 0, absent; 1, minimal; 2, mild; 3, moderate; 4, marked; and 5, severe. Qualitative assessment of various inflammatory cell types (neutrophils, macrophages, and lymphocytes) and multinucleated giant cells (MNGCs) when present were also performed. After each criterion was scored individually, the scores were totaled to generate a composite score of inflammation for each skin sample. Areas of inflammation were noted as being located in the superficial dermis, deep dermis, panniculus, or subcutis.

### Statistical analysis

Individual animal body weights were normalized to baseline, and one-way ANOVA with a Dunn’s multiple comparison test was performed to compare changes in body weights at 24, 48 and 72 h after drug administration with respect to baseline for each dosing group. Normalized body weights between dosing groups for each time point were compared using a Mann–Whitney Test. Plasma buprenorphine pharmacokinetic parameters were compared between BSR and EXR by using a Mann–Whitney Test. A paired t-test was used to compare video-recorded animal activity levels between baseline and after treatment. A P-value of less than 0.001 was defined as significant. Histopathological scores of histologic sections of BSR and EXR injection sites were compared to saline control injection sites for both acute and chronic time points using a Mann–Whitney Test with Bonferroni correction. Fibrosis and collagenolysis scores of Masson trichrome stained histologic sections of chronic BSR and EXR injection sites were compared to those of chronic saline control injection sites using a Mann–Whitney Test with Bonferroni correction. Statistical analysis was conducted using Prism software (version 8, Graphpad Software, San Diego, CA). A P-value of 0.05 or less was defined as significant.

## Supplementary Information

Supplementary Figure 1.

Supplementary Information 2.

## Data Availability

The datasets generated during and/or analyzed during the current study are available from the corresponding author on reasonable request.
