# Supplementary figures and images for: Evaluation and comparison of pharmacokinetic profiles and safety of two extended-release buprenorphine formulations in common marmosets (Callithrix jacchus)
Source: Sci Rep. 2023 Jul 22;13:11864. doi: 10.1038/s41598-023-38973-2 (PMC10363172; doi:10.1038/s41598-023-38973-2)

# Supplemental Figure 1

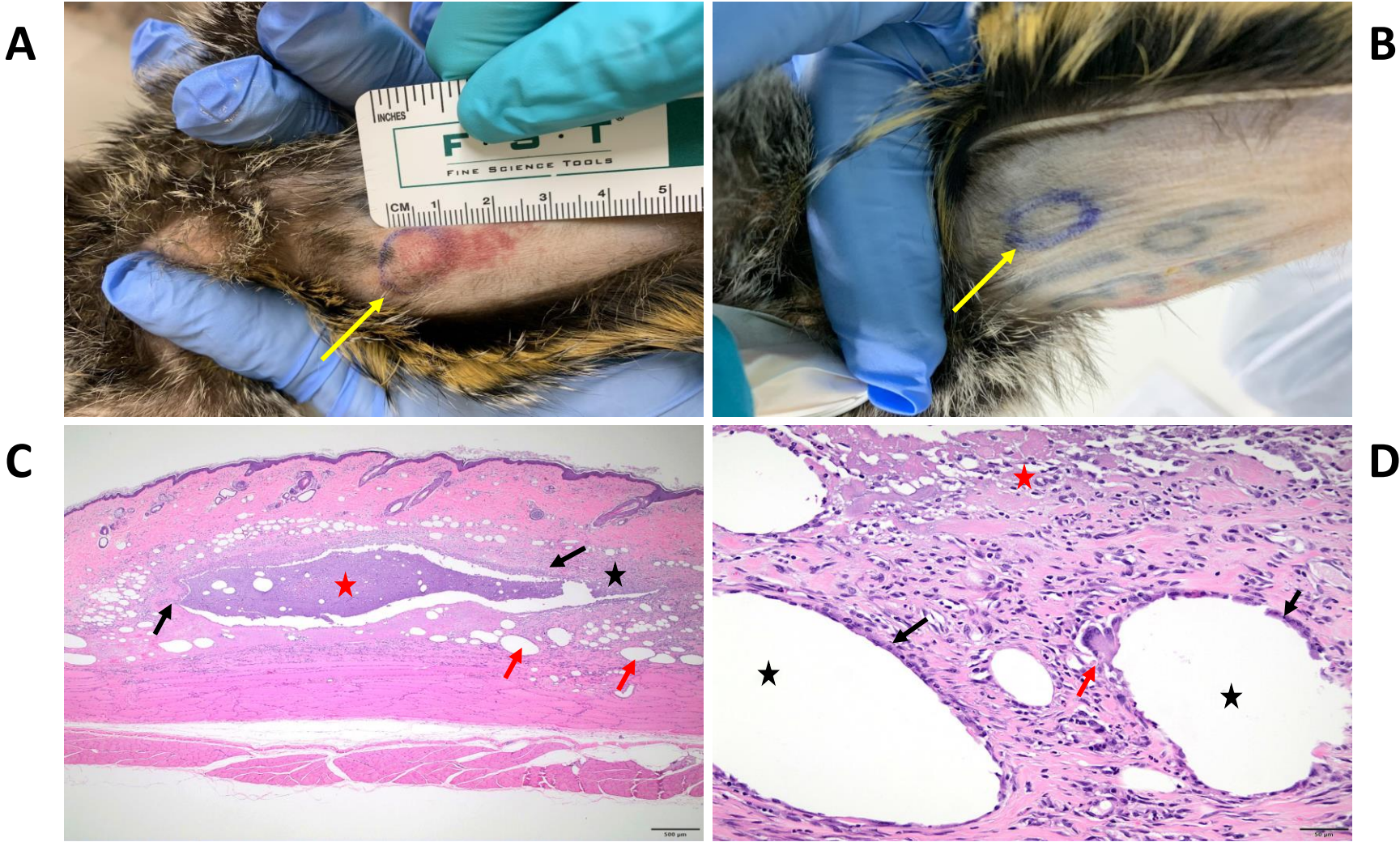

Supplement: Supplementary file 1 — Supplementary Figure 1. [file 41598_2023_38973_MOESM1_ESM.pdf]
