# Supplementary material for: Evaluation and comparison of pharmacokinetic profiles and safety of two extended-release buprenorphine formulations in common marmosets (Callithrix jacchus)
Source: Sci Rep. 2023 Jul 22;13:11864. doi: 10.1038/s41598-023-38973-2 (PMC10363172; doi:10.1038/s41598-023-38973-2)
Supplement: Supplementary file 2 — Supplementary Information 2. [file 41598_2023_38973_MOESM2_ESM.pdf]

Supplementary Information

Supplemental Figure Legends

Supplemental Figure 1. Severe injection site reaction associated with BSR (A) versus intra-animal saline control (B) observed 72 h after administration in an 11-year-old female marmoset. The BSR injection site had a round, raised, firm swelling approximately 1 cm in diameter, with erythema that extended beyond the circled region (indicated by yellow arrow in panel A) compared to contralateral saline control (indicated by yellow arrow in panel B). Representative H&E histology images of injection site reaction sections (C & D). Injection site collected 10 days after drug administration at 2x magnification (C) showing a large injection related sterile pyogranuloma (black arrows) expanding the dermis and hypodermis with a central necrotic core (red star) surrounded by inflammatory cells (black star) and oval to round clear distended spaces (red arrows) associated with injected material. 20x magnification (D) shows necrosis/inflammation (red star) and distended clear spaces (black stars) associated with injected material with an epithelioid cap (black arrows) and multinucleated giant cell (red arrow).

Supplementary Tables

Supplementary Table 1. Ataxia scoring system

Score	Ataxia
0 (No ataxia)	Normal climbing, jumping and walking
1 (Mild)	Mild stumbling
2 (Moderate)	Stumbling and uncoordinated climbing
3 (Severe)	Cannot stand, climb or jump, falls, difficulty holding up head, requires medical intervention

Supplementary Table 2. Sedation scoring system

Score	Sedation

0 (No sedation)	Vocal, resistant to hand catch, resistant to restraint, strong muscle tone
1 (Mild)	Quiet, mild resistance to hand catch, mild resistance to restraint, mild muscle relaxation
2 (Moderate)	Quiet, no resistance to hand catch, no resistance to restraint, moderate muscle relaxation, drooling
3 (Severe)	Lethargic, requires medical intervention

Supplementary Table 3. Scoring system for erythema and swelling at injection site

Score	Erythema	Swelling
0 (No abnormalities)	No visible reaction	No palpable thickening
1 (Minimal)	Pin-point scab or minimal erythema (less than 3 mm diam.)	Palpable thickening, soft, borders undefined
2 (Mild)	Mild erythema (3-6 mm diam.)	Palpable mass, firm, borders defined, within original injection site
3 (Moderate)	Moderate erythema (6-10 mm diam.) or partial thickness ulceration	Palpable mass, firm, borders defined, spread beyond original injection site
4 (Moderate to severe)	Erythema (>1 cm diam.), with lesion(s) spread beyond original injection site	Palpable mass, firm, borders defined, spread beyond original injection site
5 (Severe)	Erythema (>2 cm diam.), or full thickness ulceration, or necrosis	Palpable mass, firm, borders defined, spread beyond original injection site

Supplementary Table 4. Scoring system for needle reactivity during phlebotomy

Score	Reactivity
0	No flinch, vocalization, or head turn
1	Flinch, vocalization, or head turn

Supplementary Table 5. Extrapolated time to reach therapeutic threshold of 0.1 ng/mL buprenorphine plasma concentration

C= target therapeutic threshold	BSR 0.15 mg/kg	EXR 0.1 mg/kg	EXR 0.15 mg/kg	EXR 0.2 mg/kg
0.1 ng/mL	129.5 h or 5.4 days	64.2 h or 2.7 days	104.3 h or 4.3 days	87.7 h or 3.7 days
